# Draft genome sequences of two multidrug-resistant *Proteus mirabilis* isolates from hospital wastewater and a discharge point into Manila Bay, Philippines

**DOI:** 10.1128/mra.01411-25

**Published:** 2026-02-24

**Authors:** Shaughn Dominic M. Padon, Erica Mai T. Cabuyao, Nathaniel John Q. Cruz, Kiara Nicole D. Rodriguez, Leslie Michelle M. Dalmacio, Liza Bautista-Patacsil, Arnel B. Beltran, Aileen H. Orbecido, Joel C. Cornista

**Affiliations:** 1 Department of Biology, College of Arts and Sciences, University of the Philippines Manila 54725, Manila, Philippines; 2 Department of Biochemistry and Molecular Biology, College of Medicine, University of the Philippines Manila 54725, Manila, Philippines; 3 Department of Engineering Science, College of Engineering and Agro-Industrial Technology, University of the Philippines Los Baños, College Laguna 54729, Laguna, Philippines; 4 Department of Chemical Engineering, Gokongwei College of Engineering, De La Salle University 63148, Manila, Philippines; Fluxus Inc., Sunnyvale, California, USA

**Keywords:** multidrug-resistant *Proteus mirabilis*, sewage pumping plant, antimicrobial resistance, wastewater resistance

## Abstract

*Proteus mirabilis*, causing community and hospital-acquired urinary tract infections, is implicated in multidrug resistance. We report the draft genomes of *P. mirabilis* MA3 and *P. mirabilis* MB8, both with highly similar genomic features, recovered from different points along a hospital wastewater pathway emptying into Manila Bay, a broader coastal environment.

## ANNOUNCEMENT

*Proteus mirabilis* is a swarming, urease-positive, Gram-negative facultative anaerobe that commonly causes community- and hospital-acquired urinary tract infections (UTIs) (1–3). Ubiquitous in the environment and often multidrug-resistant, this opportunistic pathogen poses a significant clinical threat (4). Here, we describe the draft genomes of two multidrug-resistant *P. mirabilis* isolates from different hospital wastewater discharge points.

*P. mirabilis* isolate MA3 was isolated from wastewater discharged from a tertiary-level hospital in Manila, Philippines (14.577742°N, 120.985553°E), while MB8 was isolated from a sewage pumping plant in the Tondo district, Manila (14.6027°N, 120.9668°E). One-liter wastewater samples, in triplicate, were retrieved from 15 to 20 ft depth and transported at 4°C to the Microbiology Laboratory, University of the Philippines Manila (Manila, Philippines). Samples were serially diluted in 0.85% (wt/vol) saline solution, spread-plated onto nutrient agar (HiMedia, India), and incubated at 30°C for 24 h. Ten randomly selected colonies, per sampling site, were screened against 14 different commonly dispensed antibiotics. Isolates MA3 (0.500) and MB8 (0.357) were selected for whole-genome sequencing (WGS), based on their multiple antibiotic resistance (MAR) indices.

Bacterial cells were grown overnight in nutrient broth (HiMedia, India) at 30°C, without shaking. An amount of 5 mL was used to extract genomic DNA using the HiPurA Bacterial Genomic DNA Purification Kit (HiMedia), in 1.5 mL aliquots, following the manufacturer’s protocol. Concentration and purity (*A*_260_/*A*_280_) of extracted gDNA were determined using the LVis plate on FLUOstar Omega (BMG LABTECH, Germany) multi-mode microplate reader. Sequencing libraries were prepared at the DNA Sequencing Core Facility, Philippine Genome Center, University of the Philippines Diliman (Quezon City, Philippines). TruSeq DNA Nano Kit (Illumina, USA) was used for library construction using sample gDNA (MA3: 21.1 ng/µL, MB8: 23.3 ng/µL). Samples were indexed with unique barcodes and pooled for multiplexing. Sequencing was performed on the Illumina MiSeq platform using 2 × 250 bp paired-end chemistry.

Trimmomatic 0.39 (5) was used for quality control of raw reads, and SPAdes 4.2.0 (6) for *de novo* assembly. Assembly quality was evaluated using QUAST 5.2.0 (7) and gVolante 2 (8). Draft genomes were annotated using the NCBI Prokaryotic Genome Annotation Pipeline (PGAP) 6.1 (9). Taxonomic identities were determined using *k*-mer signatures (KmerFinder 3.2) (10–12) and pairwise average nucleotide identities (JSpeciesWS server) (13, 14). Multilocus sequence typing (MLST) was performed using PubMLST (15). Human pathogenic potential of the strains was predicted using PathogenFinder2 0.5.0 (16). Default parameters were implemented for all modules.

*K*-mer signatures identified both MA3 and MB8 as *P. mirabilis* (87.88% and 92.76% identity, respectively). Pairwise ANI confirmed this, showing high similarity (>98.9%) to type strain ATCC 29906^T^ and other clinical isolates (FDAARGOS_85 and FDAARGOS_67). Notably, MA3 and MB8 share 99.1% ANI, indicating they are closely related. MLST classified MB8 as sequence type (ST) 186, an emerging clonal lineage, based on 100% identity across 19 housekeeping and flagellar loci. Assembly statistics are summarized in Table 1.

**TABLE 1:** Scaffold-level assembly characteristics of *Proteus mirabilis* strains MA3 and MB8

General features	*Proteus mirabilis* MA3	*Proteus mirabilis* MB8
% Genome completeness	91.16	94.02
% Genome contamination	1.48	4.27
Paired-end reads	2 × 881,577	2 × 995,095
Genome size (Mbp)	3.9	4.0
Number of contigs	197	185
Number of scaffolds	194	183
Number of plasmids	2	0
% G+C content	38.5	38.5
Genome coverage	56.2×	59.7×
Scaffold *N*_50_ (kb)	226.7	210.7
Scaffold *L*_50_	5	6
Coding sequences (CDS)	3,617	3,675
CDS ratio	0.99	0.97
Pseudogenes	38	52
RNAs	141	135
rRNA	46	49
tRNA	81	82
ncRNA	4	4
Pathogenicity likelihood (%)	97.42 ± 1.07	96.80 ± 1.41

## Data Availability

The draft genome assembly and raw reads were deposited in NCBI GenBank under accession numbers for both MA3 (GCA_052071175.1, SRS26155767) and MB8 (GCA_052071355.1, SRR34971366) strains. All of these were deposited under BioProject PRJNA1302472.

## References

[1] Pearson MM, Rasko DA, Smith SN, Mobley HLT. 2010. Transcriptome of swarming Proteus mirabilis. Infect Immun 78:2834–2845. doi:10.1128/IAI.01222-09 20368347 PMC2876570

[2] Chen C-Y, Chen Y-H, Lu P-L, Lin W-R, Chen T-C, Lin C-Y. 2012. Proteus mirabilis urinary tract infection and bacteremia: risk factors, clinical presentation, and outcomes. J Microbiol Immunol Infect 45:228–236. doi:10.1016/j.jmii.2011.11.007 22572004

[3] Ma W-Q, Han Y-Y, Zhou L, Peng W-Q, Mao L-Y, Yang X, Wang Q, Zhang T-J, Wang H-N, Lei C-W. 2022. Contamination of Proteus mirabilis harbouring various clinically important antimicrobial resistance genes in retail meat and aquatic products from food markets in China. Front Microbiol 13:1086800. doi:10.3389/fmicb.2022.1086800 36590410 PMC9802577

[4] Drzewiecka D. 2016. Significance and roles of Proteus spp. bacteria in natural environments. Microb Ecol 72:741–758. doi:10.1007/s00248-015-0720-6 26748500 PMC5080321

[5] Bolger AM, Lohse M, Usadel B. 2014. Trimmomatic: a flexible trimmer for Illumina sequence data. Bioinformatics 30:2114–2120. doi:10.1093/bioinformatics/btu170 24695404 PMC4103590

[6] Bankevich A, Nurk S, Antipov D, Gurevich AA, Dvorkin M, Kulikov AS, Lesin VM, Nikolenko SI, Pham S, Prjibelski AD, Pyshkin AV, Sirotkin AV, Vyahhi N, Tesler G, Alekseyev MA, Pevzner PA. 2012. SPAdes: a new genome assembly algorithm and its applications to single-cell sequencing. J Comput Biol 19:455–477. doi:10.1089/cmb.2012.0021 22506599 PMC3342519

[7] Gurevich A, Saveliev V, Vyahhi N, Tesler G. 2013. QUAST: quality assessment tool for genome assemblies. Bioinformatics 29:1072–1075. doi:10.1093/bioinformatics/btt086 23422339 PMC3624806

[8] Nishimura O, Hara Y, Kuraku S. 2017. gVolante for standardizing completeness assessment of genome and transcriptome assemblies. Bioinformatics 33:3635–3637. doi:10.1093/bioinformatics/btx445 29036533 PMC5870689

[9] Tatusova T, DiCuccio M, Badretdin A, Chetvernin V, Nawrocki EP, Zaslavsky L, Lomsadze A, Pruitt KD, Borodovsky M, Ostell J. 2016. NCBI prokaryotic genome annotation pipeline. Nucleic Acids Res 44:6614–6624. doi:10.1093/nar/gkw569 27342282 PMC5001611

[10] Hasman H, Saputra D, Sicheritz-Ponten T, Lund O, Svendsen CA, Frimodt-Møller N, Aarestrup FM. 2014. Rapid whole-genome sequencing for detection and characterization of microorganisms directly from clinical samples. J Clin Microbiol 52:139–146. doi:10.1128/JCM.02452-13 24172157 PMC3911411

[11] Larsen MV, Cosentino S, Lukjancenko O, Saputra D, Rasmussen S, Hasman H, Sicheritz-Pontén T, Aarestrup FM, Ussery DW, Lund O. 2014. Benchmarking of methods for genomic taxonomy. J Clin Microbiol 52:1529–1539. doi:10.1128/JCM.02981-13 24574292 PMC3993634

[12] Clausen PTLC, Aarestrup FM, Lund O. 2018. Rapid and precise alignment of raw reads against redundant databases with KMA. BMC Bioinformatics 19:307. doi:10.1186/s12859-018-2336-6 30157759 PMC6116485

[13] Richter M, Rosselló-Móra R, Oliver Glöckner F, Peplies J. 2016. JSpeciesWS: a web server for prokaryotic species circumscription based on pairwise genome comparison. Bioinformatics 32:929–931. doi:10.1093/bioinformatics/btv681 26576653 PMC5939971

[14] Richter M, Rosselló-Móra R. 2009. Shifting the genomic gold standard for the prokaryotic species definition. Proc Natl Acad Sci USA 106:19126–19131. doi:10.1073/pnas.0906412106 19855009 PMC2776425

[15] Jolley KA, Bray JE, Maiden MCJ. 2018. Open-access bacterial population genomics: BIGSdb software, the PubMLST.org website and their applications. Wellcome Open Res 3:124. doi:10.12688/wellcomeopenres.14826.1 30345391 PMC6192448

[16] Ferrer Florensa A, Almagro Armenteros JJ, Kaas RS, Conradsen Clausen PTL, Nielsen H, Rost B, Aarestrup FM. 2025. Whole-genome prediction of bacterial pathogenic capacity on novel bacteria using protein language models, with PathogenFinder2. bioRxiv. doi:10.1101/2025.04.12.648497 PMC13218381 41863303

